# Spontaneous Fragmentation and the Disappearing JJ Stent: A Case Series and Review of the Literature

**DOI:** 10.7759/cureus.93141

**Published:** 2025-09-24

**Authors:** Khaled Ghanem, Shady Hegab, Junaid Masood

**Affiliations:** 1 Department of Urology, Barking, Havering and Redbridge University Hospitals National Health Service (NHS) Trust, London, GBR; 2 Department of Radiology, Barking, Havering and Redbridge University Hospitals National Health Service (NHS) Trust, London, GBR

**Keywords:** complication, endo urology, prevention, spontaneous fragmentation, ureteric stent

## Abstract

When double J urinary stents are “forgotten,” their constituent polymers undergo mechanical and chemical degradation. This process can lead to potentially serious complications, such as encrustation or fragmentation. Fragmented and degraded stents are often more difficult to remove, frequently requiring complex surgical procedures. We present two cases of ureteric stents that were left in place for more than three years. In one case, a complicated ureteroscopy was required to remove the stent in numerous pieces. In the other case, the stents fragmented over time and completely disappeared; the patient denied experiencing stenturia. We review the evidence to identify the potential mechanisms leading to stent fracture and suggest possible preventive strategies.

## Introduction

Double J (DJ) ureteral stents are widely used in urological practice for the management of ureteric obstruction due to urolithiasis, strictures, postoperative oedema, or extrinsic compression from malignancies or retroperitoneal fibrosis [1,2]. While highly effective, their use is associated with well-recognised morbidity, including flank pain, haematuria, lower urinary tract symptoms, and urinary tract infections [3,4].

DJ stents are typically made of biocompatible materials such as silicone, polyurethane, hydrophilic-coated copolymers (e.g., Percuflex® [Boston Scientific, Massachusetts, USA], Cflex® [Cook Medical, Bloomington, USA], and Silitek®), and occasionally metallic or biodegradable polymers. Silicone stands out with its softness, flexibility, and low encrustation rate, whereas copolymers are more biocompatible and easily placed. Metallic stents offer durability for long-term drainage, and biodegradable variants reduce the need for retrieval by gradually dissolving in situ; some are currently still under investigation [5]. They earned the name “double J” due to their curled ends securing placement in the renal pelvis and bladder while allowing urinary drainage. Recommended indwelling times typically range from short- to intermediate-term (up to 6-12 weeks), with specialised long-term stents designed for up to several months of use; exceeding these intervals substantially elevates the risk of complications such as encrustation, migration, obstruction, or fragmentation [6,7].

The situation becomes more problematic when stents are inadvertently “forgotten” or retained for prolonged periods. Extended indwelling time is the most significant risk factor for serious complications such as encrustation, migration, obstruction, and even life-threatening urosepsis [3,4,8]. Encrustation in particular can make removal technically difficult, often requiring complex or staged endourological procedures [1,4].

An even rarer but clinically important complication is spontaneous stent fragmentation, sometimes resulting in “stenturia,” where patients pass fragments of the stent per urethra [8,9]. Only a limited number of cases have been reported in the literature, with fragmentation occurring as early as three weeks post-insertion in some instances [8,10,11]. Proposed mechanisms include material fatigue, prolonged urinary exposure, biofilm formation, infection, and repeated mechanical stress on the stent [2,12,13]. Polyurethane stents appear particularly prone to this phenomenon [13].

Spontaneous rupture not only complicates stent retrieval but can also serve as a nidus for infection and stone formation, further increasing patient morbidity [5,11,14]. Awareness of this rare event is therefore crucial, as delayed recognition may lead to significant clinical consequences.

In this report, we present two cases of spontaneous fragmentation of long-standing forgotten DJ stents. We also review the available literature to provide insights into this rare complication and highlight preventive strategies, including timely follow-up, patient education, and stent tracking systems, as well as emerging innovations such as biodegradable stents [15].

## Case presentation

Case 1

**Figure 1:**
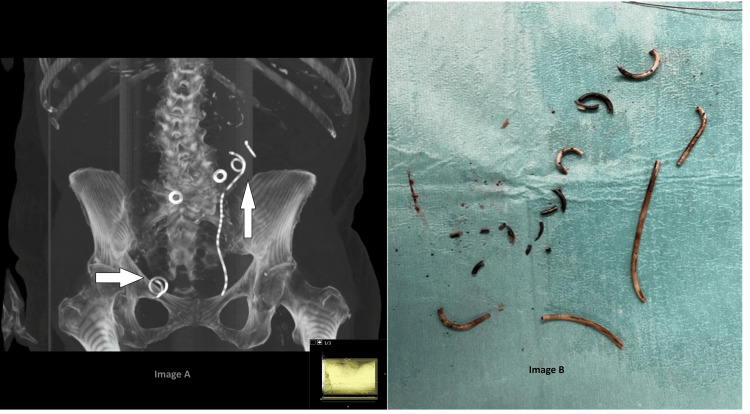
Image A shows a MIP (maximum intensity projection image) from a coronal section of a CT abdomen and pelvis showing a modified bone window with pieces of fragmented stent coils in the kidney (vertical arrow) and bladder (horizontal arrow), and Image B shows the stent removed in numerous pieces. (A) Maximum intensity projection MIP CT image of the abdomen and pelvis with a modified bone window showing multiple fragmented coils of a double J ureteral stent in the kidney (vertical arrow) and bladder (horizontal arrow). (B) Intraoperative photograph demonstrating the stent retrieved in multiple fragmented pieces.

An 84-year-old woman with a history of rheumatoid arthritis, nephrolithiasis, and chronic kidney disease (CKD) underwent antegrade insertion of a left-sided 8F multilength polyurethane JJ stent to relieve renal obstruction following a Hartmann’s procedure and excision of a colo-uterine fistula. Having been lost to follow-up for over three and a half years due to non-attendance, she presented with urinary sepsis. CT KUB revealed that the stent had fragmented into numerous pieces (Figure 1). A lengthy (161-minute) combined rigid and flexible ureteroscopic approach was required to remove the stent. The procedure was complicated by further fragmentation during removal, with the stent ultimately retrieved in multiple pieces.

Case 2

**Figure 2:**
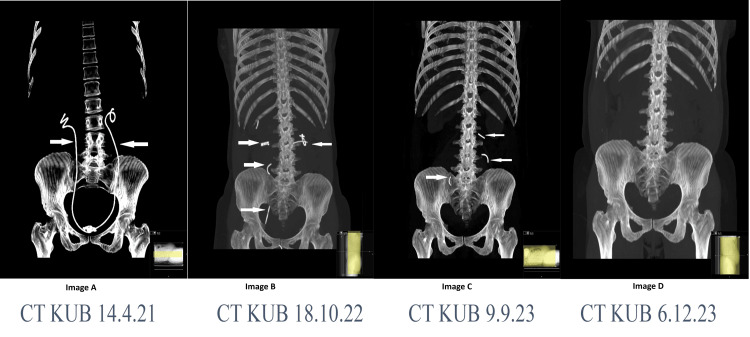
MIP (maximum intensity projection image) images from coronal sections of a CT kidney, ureter and bladder with a modified bone window showing gradual spontaneous fragmentation and disappearance of bilateral double J stents over time. Figure 2. Serial maximum intensity projection (MIP) CT images of the kidneys, ureters, and bladder (KUB) demonstrating gradual spontaneous fragmentation and disappearance of bilateral double J stents over time. (A) April 14, 2021: Intact bilateral ureteral stents in situ (white arrows) seen extending from the kidneys to the urinary bladder. (B) October 18, 2022: Multiple fragmented stent pieces along the ureters (white arrows) are seen within the kidneys and the right ureter, with the disappearance of most of the stent fragments from the left ureter and the bladder. (C) September 9, 2023: Further fragmentation with only small fragments remaining (white arrows) within the left kidney and proximal right and left ureters. (D) December 6, 2023: Complete disappearance of both stents

A 51-year-old woman with a history of myotonic dystrophy, obesity, pulmonary embolism, and obstructive sleep apnoea underwent bilateral antegrade insertion of 8F multilength polyurethane double J stents to relieve ureteric obstruction caused by a very large uterine fibroid. Non-compliance with planned follow-up for gynaecological surgery, combined with delays in care due to the COVID-19 pandemic, resulted in the stents remaining in situ for more than three years. CT imaging in April 2021 demonstrated intact stents (Figure 2); however, subsequent scans showed progressive fragmentation and disappearance of stent segments. By December 2023, CT imaging revealed no stents in situ. The patient denied experiencing stenturia.

Table 1 shows characteristics, management, and outcomes of the two cases.

**Table 1:** Characteristics, management, and outcomes of two cases of long-term indwelling DJ stent fragmentation.

Case	Age / Sex	Indication for Stent	Stent Material / Type	Indwelling Time	Fragmentation / Location	Management	Outcome
1	84 / F	Renal obstruction post-Hartmann’s procedure and colo-uterine fistula excision	8F multilength polyurethane DJ stent	~3.5 years	Multiple fragments (left ureter, kidney, bladder)	Combined rigid and flexible ureteroscopy (161 min), stent retrieved in pieces	Resolution of infection after removal
2	51 / F	Bilateral ureteric obstruction due to large uterine fibroid	8F multilength polyurethane DJ stents (bilateral)	>3 years	Progressive fragmentation and eventual disappearance; no stents seen by Dec 2023	Conservative (no retrieval, spontaneous loss)	Remained asymptomatic, no stenturia reported

## Discussion

Polyurethane stents, such as those used in these patients, are widely employed due to the low cost and versatility of the material [16]. Double J stents have well-documented early and late complications [1]. Spontaneous fragmentation of stents is rare and is classified as grade 3 on the Clavien classification of surgical complications [16].

The ideal stent material would be biologically inert, chemically stable in urine, resistant to encrustation and infection, capable of maintaining long-term urinary flow, and cost-effective [12]. However, such material does not yet exist. Most commercially available ureteric stents are composed of polyurethane, Percuflex, or silicone. Each offers a balance between flexibility and durability, but none are completely resistant to degradation [2].

Theories explaining stent fragmentation primarily focus on material fatigue and chemical degradation. Over time, mechanical stress from ureteric peristalsis and urine flow leads to a loss of tensile strength and flexibility, predisposing the stent to microfractures [1,2,12]. Although originally thought to occur only when stents remained in situ for more than one year, El Mostapha and colleagues suggested that stents left for more than six months have a higher risk of fragmentation due to polymer degradation [11]. El-Faqih et al. reported a fragmentation rate of 0.3% for stents retained for longer than three months [1], but cases have been documented as early as three weeks post-insertion [10]. Reported rates in the literature vary between 0.3% and 10% [1,14].

Exposure to a harsh urinary environment changes stent material from ductile to brittle [6]. Polyurethane has relatively poor biodurability, and ageing can result in early mechanical failure. Stent lengths longer than appropriate for the patient can increase mechanical stress and further raise the risk of fragmentation [16]. Chua reported that polyurethane stents are four times more likely to fragment than those made from silicone or other biomaterials [16]. Fractures tend to occur at the holes or drainage points along the stent [11].

Long-term exposure to urine, particularly in the presence of infection, can catalyse chemical degradation of the polymer [8]. Specific bacterial enzymes, certain urinary constituents (including minerals), and acidic environments can make urine more hostile [8,13]. Inflammatory reactions may also initiate and promote degradation [11]. Ilker found significant numbers of leucocytes in the urine of affected patients, with or without infection, suggesting that lysosomal enzyme release from these cells may contribute to stent depolymerisation [17]. Mineral deposits or encrustations that form on the stent can further increase brittleness and the risk of fracture [7].

While stent placement generally suppresses ureteric peristalsis and is unlikely to directly cause fragmentation, residual contractile activity may contribute to distal propulsion of fragments, similar to the passage of ureteric stones or stent migration [3,4]. This mechanism may explain the occurrence of stenturia in some patients.

To address the limitations of conventional double J stents, biodegradable ureteric stents have been developed. These are designed to degrade safely within the urinary tract, eliminating the need for removal. Although promising, current prototypes remain unreliable, with unpredictable degradation rates and fragment sizes, inflammatory responses, and mechanical weaknesses limiting their widespread use [15].

For now, closer monitoring and improved patient education are essential. Timely stent exchange or removal, correct stent length selection, and safeguards such as electronic stent registries to track patients are vital in reducing serious complications such as encrustation and fragmentation. Additional preventive measures include dietary modification to reduce urinary hostility, adequate hydration, prevention of urinary tract infection, and the possible use of potassium citrate to alkalinise the urine.

Further research into more durable or predictable biodegradable alternatives is needed to reduce the risks associated with long-term stent use and to improve patient outcomes.

## Conclusions

Spontaneous fragmentation of forgotten double J stents is a rare but potentially serious complication that can pose significant challenges in management. The risk increases with prolonged dwell time, particularly beyond six months, and is influenced by both mechanical and chemical degradation of stent polymers. Preventive strategies, such as timely stent exchange or removal, use of appropriate stent length, patient education, and implementation of electronic stent registries, are essential to minimise this risk. Continued research into more durable materials or reliable biodegradable alternatives is necessary to improve patient safety and outcomes.
